# Temporal Muscle Thickness and Sarcopenia Components in Healthy Adults, Validated through Allgeun Diagnostic Tool

**DOI:** 10.3390/healthcare12101012

**Published:** 2024-05-14

**Authors:** Kang Min Park, Ho-Joon Lee, Bong Soo Park, Jin-Hong Wi, Yong-Uk Kwon, Won Hee Lee, Dong Ah Lee, Jinseung Kim

**Affiliations:** 1 Department of Neurology, Haeundae Paik Hospital, Inje University College of Medicine, Busan 48108, Republic of Korea; smilepkm@hanmail.net (K.M.P.); peony1003@gmail.com (D.A.L.); 2 Department of Radiology, Haeundae Paik Hospital, Inje University College of Medicine, Busan 48108, Republic of Korea; 3 Department of Internal Medicine, Haeundae Paik Hospital, Inje University College of Medicine, Busan 48108, Republic of Korea; h00245@paik.ac.kr; 4 Department of Thoracic and Cardiovascular Surgery, Busan Paik Hospital, Inje University College of Medicine, Busan 47392, Republic of Korea; wiccy@hanmail.net; 5 Department of Orthopedic Surgery, Busan Paik Hospital, Inje University College of Medicine, Busan 47392, Republic of Korea; kyw2848@hanmail.net; 6 Department of Neurosurgery, Busan Paik Hospital, Inje University College of Medicine, Busan 47392, Republic of Korea; ywh9868@hanmail.net; 7 Department of Family Medicine, Busan Paik Hospital, Inje University College of Medicine, Busan 47392, Republic of Korea

**Keywords:** sarcopenia, temporal muscle thickness, calf circumference, physical performance

## Abstract

Sarcopenia, characterized by progressive muscle loss and functional decline, poses significant risks, including falls, impaired daily activities, and increased mortality. We developed Allgeun, a novel device that measures handgrip strength, muscle mass, and physical performance. This study aimed to investigate whether temporal muscle thickness (TMT) could be used as a sarcopenia marker and to evaluate the usability of Allgeun. This prospective study enrolled 28 participants without medical or neurological disorders. They underwent three-dimensional T1-weighted imaging using a 3 Tesla magnetic resonance imaging scanner. TMT was measured based on T1-weighted images by a board-certified neuroradiologist. Allgeun was used to measure the following three key components of sarcopenia: muscle strength (handgrip strength), muscle mass (calf and thigh circumference), and physical performance (five times the chair stand test). Correlation analysis was conducted between TMT and the results of the handgrip strength, calf and thigh circumferences, and chair stand tests. There were moderate positive correlations between TMT and calf circumference (r = 0.413, *p* = 0.029), thigh circumference (r = 0.486, *p* = 0.008), and handgrip strength (r = 0.444, *p* = 0.018). However, no significant correlation was observed between TMT and physical performance (r = −0.000, *p* = 0.998). Our findings underscore TMT’s potential as an indicator of sarcopenia, particularly regarding muscle mass and strength. Additionally, we demonstrated that the new device, Allgeun, is useful for screening and diagnosing the severity of sarcopenia.

## 1. Introduction

Sarcopenia is a progressive and generalized skeletal muscle condition characterized by the gradual loss of muscle mass and function, resulting in significant impairment of overall physical performance and strength [1]. According to a recent systematic review and meta-analysis, the current estimated prevalence of sarcopenia among older adults (aged > 60 years) globally falls within the range of 10% to 16% [2]. The estimated prevalence of sarcopenia among community dwellers aged 60 years and older is around 11% in men and 9% in women, while nursing home residents exhibit higher rates, affecting approximately half of elderly men and one third of elderly women [3]. It is inherently linked with an elevated susceptibility to falls, reduced physical function, increased frailty, and a heightened likelihood of mortality, underscoring the multifaceted impact of sarcopenia on an individual’s health and well-being [4]. Sarcopenia is intricately connected to a heightened risk of fractures, hindering the capacity to execute routine daily activities; furthermore, its associations extend to cardiac and respiratory diseases, cognitive impairment, the emergence of mobility issues, and a substantial contribution to an overall diminished quality of life for affected individuals [5,6,7,8,9,10,11].

Sarcopenia is diagnosed upon the presence of observable evidence indicating a decline in both muscle quantity and quality. The severity of sarcopenia is assessed through concurrent manifestations, encompassing not only low muscle strength but also the compromised aspects of muscle quantity/quality, coupled with impaired physical performance, thereby providing a comprehensive evaluation of the condition [4]. The initial diagnosis of sarcopenia includes the evaluation of muscle strength, particularly grip strength [12]. Subsequent diagnostic procedures involve the quantification of muscle mass, primarily gauged through dual-energy X-ray absorptiometry (DXA) to assess lean mass; additionally, other methodologies, including bioelectrical impedance analysis (BIA) and computed tomography (CT) scans, may be employed to offer a comprehensive evaluation of muscle composition [13]. However, the comprehensive assessment of skeletal muscle mass and function necessitates additional examinations, potentially resulting in heightened radiation exposure and extended healthcare expenses, thereby emphasizing the need for a balanced consideration of diagnostic approaches in the evaluation of sarcopenia. The measurement of calf circumference proves to be an effective diagnostic tool, exhibiting moderate-to-high sensitivity and specificity in accurately predicting the presence of sarcopenia or indicating low skeletal muscle mass, underscoring its utility as a reliable and accessible indicator in clinical assessments [14,15]. In a recent study, it was found that the measurement of calf circumference surpasses the skeletal mass index based on BIA in terms of effectiveness in assessing muscle mass, displaying notably higher specificity, while concurrently upholding comparable sensitivity levels, highlighting its potential as a superior diagnostic measure in the evaluation of skeletal muscle mass [16].

Recent scientific investigations have revealed a significant correlation between temporal muscle thickness (TMT) and the risk of sarcopenia, with routine brain magnetic resonance imaging (MRI) emerging as a valuable tool for estimating muscle mass. Notably, TMT has been explored as an innovative surrogate indicator, offering promising insights into the identification of sarcopenia risk among individuals with neurological conditions, thereby enhancing our understanding of its potential applicability in diverse clinical contexts [17,18]. Prior investigations have consistently demonstrated a strong and reliable connection between TMT, handgrip strength, and skeletal muscle mass, thereby solidifying TMT’s role as a dependable and comprehensive indicator for the thorough assessment of sarcopenia, underscoring its multifaceted utility in gauging muscular health [17,18,19]. TMT can be conveniently and efficiently assessed through standard brain CT or MRI scans, providing an optimal and accessible methodology for investigating sarcopenia. Additionally, this approach facilitates retrospective analysis by utilizing previously acquired imaging, enhancing its practicality and versatility in retrospective studies on muscular health. Previous studies demonstrating an association between TMT and sarcopenia primarily focused on patients with neurological disorders. One study demonstrated established correlations between handgrip strength and lumbar skeletal muscle in patients with brain metastasis, and another study examined the association between TMT and strength, assistance in walking, rising from a chair, climbing stairs, and falls (SARC-F) questionnaire scores in patients with ischemic stroke [17,18,19]. The limitations of these earlier studies become apparent, given their exclusion of healthy individuals as subjects and the absence of direct measurements of calf circumference or assessments of physical performance, thereby emphasizing the need for more comprehensive investigations to provide a thorough understanding of sarcopenia across diverse populations. Moreover, a notable gap exists in the current body of research, as there is a deficiency of studies directly comparing the constituent elements of sarcopenia, encompassing handgrip strength, calf circumference, and physical performance, with TMT within the specific context of sarcopenia, underscoring the necessity for comprehensive investigations to elucidate the intricate relationships among these parameters.

In this study, to assess the correlation between TMT and the diagnostic components of sarcopenia, we used handgrip strength for muscle strength, calf and thigh circumference for muscle mass, and the five-time chair stand test for physical performance. Although there are slight differences between the guidelines of the Asian Working Group for Sarcopenia (AWGS) and the European Working Group on Sarcopenia in Older People (EWGSOP), the AWGS 2019 guidelines recommend the five-time chair stand test for evaluating physical performance [20]. Due to the stepwise diagnostic approach of sarcopenia, confirming both diminished muscle strength and reduced muscle mass, as well as considering compromised physical performance for severe cases, diagnosing sarcopenia becomes challenging [4]. In response to this difficulty, we introduced an innovative diagnostic device, Allgeun, seamlessly integrating the three sarcopenia components into a single, portable, and user-friendly solution. As some studies have demonstrated the relevance of thigh muscle thickness or circumference in sarcopenia, we measured thigh muscle thickness to compare and assess its association with sarcopenia [21,22].

This study aimed to investigate TMT viability as a sarcopenia marker and evaluate the usability of the new Allgeun device. 

## 2. Methods

### 2.1. Participants

This study, which was conducted in accordance with the ethical standards of the Declaration of Helsinki, received approval from the Institutional Review Board of Inje University Busan Paik Hospital (approval number: 2022-11-027). While the requirement for written informed consent was waived due to the retrospective nature of the study, in the case of the prospective study, explicit informed consent was diligently obtained from each participant prior to their involvement. Additionally, the research protocol obtained official approval from the institutional review board of our center, demonstrating meticulous adherence to ethical standards in research conduct. We prospectively enrolled the participants between August 2023 and December 2023. Enrollment in the study was limited to individuals who did not present any discernible medical or neurological disorders, emphasizing a stringent selection criterion focused on recruiting participants without pre-existing health conditions that could potentially confound the research findings. Furthermore, none of the participants exhibited any structural brain lesions upon thorough examination of their MRI scans.

### 2.2. MRI Acquisition

All participants lied down in the supine position with their mouths slightly closed. All participants underwent three-dimensional (3D) T1-weighted imaging using a 3 Tesla MRI scanner with a 32-channel head coil (AchievaTx, Phillips Healthcare, Best, Netherlands). The 3D T1-weighted image was acquired in the sagittal plane using a turbo-field echo sequence with the following parameters: TI = 1300 ms, repetition time/echo time = 8.6/3.96 ms, flip angle = 8°, and an isotropic voxel size of 1 mm³. We measured TMT using this 3D T1-weighted image. In addition, 3D fluid attenuated inversion recovery images (FLAIR) were taken in all participants to examine the presence or absence of structural lesions in their brain.

### 2.3. TMT Measurement

**Figure 1:**
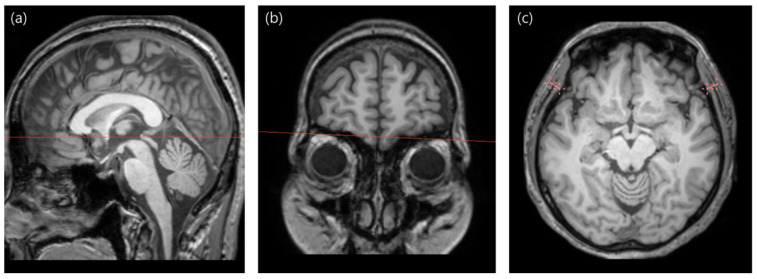
Illustration of temporal muscle thickness measurement procedure. (**a**) Images were reformatted to the axial plane parallel to the anterior commissure–posterior commissure line. (**b**) The view was navigated to the orbital roof level. (**c**) Thickness measurements of the temporalis muscle were taken on both sides using the Sylvian fissure as the anterior–posterior reference point.

TMT measurements were meticulously conducted on both the right and left sides by a highly qualified and board-certified radiologist (H. J. L., with 9 years of subspecialty experience in neuroradiology). The acquired images underwent meticulous reformatting, aligning with an axial plane parallel to the anterior commissure–posterior commissure line to guarantee optimal visualization and precision in the anatomical assessment. TMT was then measured perpendicular to the long axis of the temporal muscle using the orbital roof and Sylvian fissure as landmarks. Image reformatting and measurements were performed using 3D Slicer (version 5.4.0, https://www.slicer.org (accessed on 2 January 2024)) [23,24]. The measurements for each side were averaged and used for further analysis. Figure 1 illustrates the TMT measurement procedure.

### 2.4. A Simple Questionnaire to Rapidly Diagnose Sarcopenia (SARC-F)

All participants underwent the administration of the Korean version of the SARC-F questionnaire as a screening tool for assessing the risk of sarcopenia. This questionnaire comprehensively evaluated various components related to strength, walking assistance, rising from a chair, climbing stairs, and the occurrence of falls. The Korean SARC-F questionnaire consists of five objective questions, each assigned a score ranging from 0 to 2, enabling a total score within the range of 0 to 10. The questionnaire is directly self-reported by the participants.

### 2.5. Allgeun: Device for Measurement

**Figure 2:**
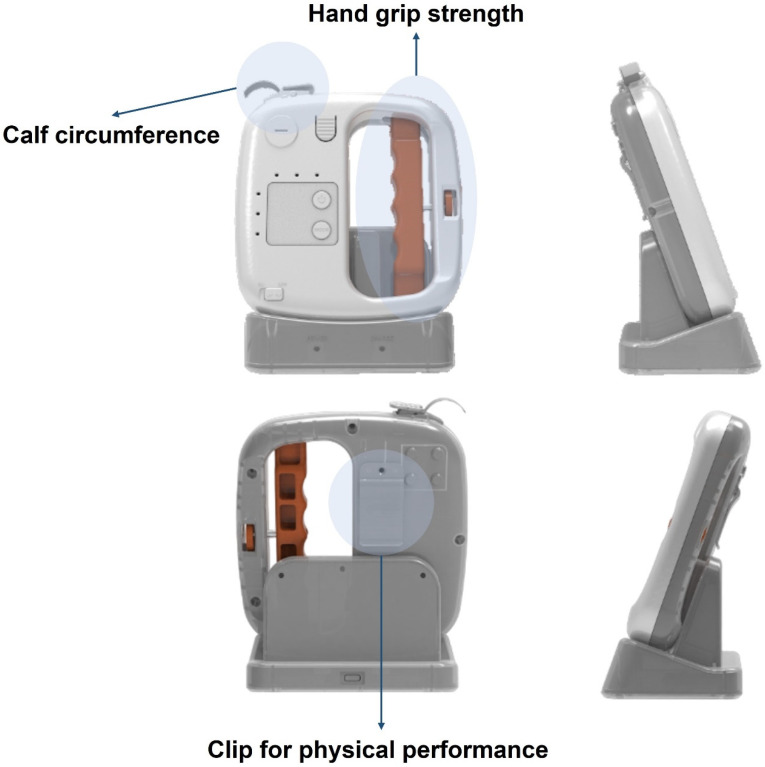
Photographs of Allgeun, including front, rear, left, and right views. In the front view, the arrows indicate calf circumference and handgrip strength, both essential for diagnosing sarcopenia. In contrast, in the rearview, the arrow indicates the clip used to measure physical performance, which is necessary for assessing the severity of sarcopenia. The device is designed to be portable and user-friendly, facilitating its straightforward utilization in primary care settings.

Allgeun was innovatively engineered as a singular device tailored to precisely measure the three key components of sarcopenia: muscle strength (assessed through handgrip strength), muscle mass (evaluated via calf and thigh circumference measurements), and physical performance (quantified using the five-time chair stand test). The fundamental objective behind the design of this device is to ensure user-friendliness within commonplace medical settings, prioritizing simplicity and avoiding unnecessary complexity and excessive size for enhanced practicality and accessibility. For the assessment of grip strength, we employed a load cell featuring the HX711 chip from Aia Semicon, skillfully designed to measure values within the 0–60 kg range with an impressive precision of 0.1 kg. The spring-type dynamometer (Smedley, Tokyo, Japan) and hydraulic-type (Jamar, Lafayette, IN, USA) are the most frequently utilized devices in Asia [25]. According to AWGS 2019 recommendations, either device can be employed for diagnosing sarcopenia, provided that standard protocols for the specific model are adhered to. As per the EWGSOP, the Jamar dynamometer stands as a validated and widely adopted tool for measuring grip strength, although exploration into the utilization of other brands is ongoing [26]. To address the drawbacks of cumbersome and heavy dynamometers, in this study, we employed a load cell type compatible with the Allgeun device. A load cell converts force into an electrical signal that can be measured, with the signal changing proportionally to the force applied. To gauge calf and thigh circumference, a 12-bit resolution AS5600 magnetic encoder was utilized, integrating a chip from ams OSRAM, effectively converting rotational values into length units for accurate display. In the execution of the five-time chair stand test, the MP6050 3 Axis Accelerometer Gyroscope Module, equipped with a chip from TDK InvenSense, was utilized to adeptly detect participant movements and precisely measure the elapsed time. The measurement of time units was executed with precision, capturing intervals up to 0.1 s. This device was intentionally crafted in a compact single-device form, and its physical appearance is illustrated in detail in Figure 2, providing a visual representation of its design and functionality.

### 2.6. Muscle Mass, Strength, and Physical Performance Measurement

The measurement of handgrip strength involved positioning the participant in an upright seated posture with their back against the chair and feet firmly planted on the floor. During the assessment, the participant’s shoulder was meticulously positioned in adduction with a neutral rotation, the elbow was systematically flexed at an exact angle of 90°, and the forearm assumed a precisely controlled neutral position, all while ensuring the wrist was carefully extended within the specified range of 0 to 30 degrees of extension [27]. Participants, adhering to the instructions provided by the test administrator, performed a maximal force squeeze on the Allgeun dynamometer, utilizing each hand individually. Three measurements, with a meticulous interval of at least 30 s between each, were systematically acquired for both the left and right sides during the handgrip-strength assessment. The recorded handgrip strength presented in the results is representative of the highest value attained from this series of measurements, ensuring a comprehensive evaluation of participants’ maximal force capabilities for precise and clinically relevant outcomes. The five-time chair stand test assesses the duration for an individual to complete five cycles of standing up and sitting down from a seated position, requiring the person to sit on a chair with arms crossed over their chest and back against the chair [28]. A chair with a standard height of 0.43 m and without armrests was used. In conjunction with this, the Allgeun device was strategically employed by positioning it on the anterior part of the thigh using a secure thigh band, ensuring stability and accuracy during the assessment. The Allgeun device, equipped with a gyroscope sensor, automated the process by recording and measuring the time in 0.1 s increments. This measurement was initiated from the moment the participant stood up and continued until the fifth sitting moment, providing a detailed and precise analysis of the entire chair stand test sequence. This sensor detected both vertical and forward–backward movements. The data were measured twice to record a prompt value. Calf circumference was assessed by placing a tape measure around the widest section of the calf while participants were seated, without compressing the subcutaneous tissue. This ensured that the calf was perpendicular to the thigh, and measurements were recorded to the nearest millimeter [29]. Thigh circumference measurement was conducted by assessing the circumference at the thinnest point of the anterior one-third of the thigh. Similar to calf circumference, the measurement was conducted without compressing the subcutaneous tissue. Participants, in a comfortable state, had their calf and thigh circumferences measured on both legs by the instructor. The larger values were recorded for both the calf and thigh measurements. The recorded measurements were automatically inputted into Allgeun and stored in the application.

### 2.7. Statistical Analysis

The Pearson correlation test, a widely recognized statistical method for examining associations between variables, was utilized in this study to analyze the correlations between TMT and various parameters, including calf circumference, thigh circumference, handgrip strength, and physical performance. All statistical analyses were performed using the MedCalc^®^ software (version 20.014, MedCalc Software, Ostend, Belgium; accessible at https://www.medcalc.org (accessed on 23 January 2024); 2021). For determining statistical significance, the predefined threshold was set at a *p*-value less than 0.05.

## 3. Results

### 3.1. Participants

A total of 28 participants were enrolled in the study, and a depiction of the clinical characteristics is presented in Table 1. The mean age of the participants was 38.1 years. Among the 28 participants, 10 men accounted for 35.7% of the total. The mean calf circumference was 37.3 cm, while the mean thigh circumference was 43.5 cm. The mean handgrip strength was 28.7 kg, and the mean physical performance, as indicated by the five-time chair stand test, was measured at 5.5 s. The mean temporal muscle thickness (TMT) was measured at 9.7 mm. When verified with the Shapiro–Wilk test, TMT was confirmed to follow a normal distribution with W = 0.9603 (*p* = 0.354). All participants in the study had a score of zero, indicating normalcy, on the comprehensive SARC-F questionnaire assessment [30].

### 3.2. Correlation Analysis between Muscle Mass/Strength and TMT

**Figure 3:**
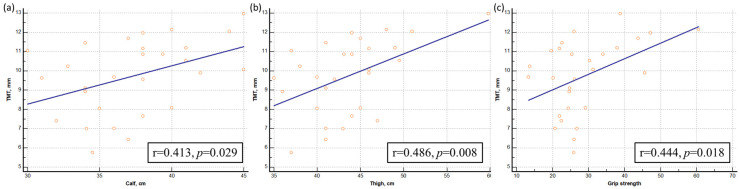
Correlation analysis between muscle mass/strength and temporal muscle thickness. The figures show moderate positive correlations between (**a**) calf circumference (r = 0.413, *p* = 0.029), (**b**) thigh circumference (r = 0.486, *p* = 0.008), (**c**) handgrip strength (r = 0.444, *p* = 0.018), and temporal muscle thickness.

Moderate positive correlations were evident among the measured variables, including calf circumference (r = 0.413, *p* = 0.029) (a), thigh circumference (r = 0.486, *p* = 0.008) (b), handgrip strength (r = 0.444, *p* = 0.018), and TMT (Figure 3). However, it is noteworthy that no significant correlation was observed between physical performance and TMT (r = −0.000, *p* = 0.998).

## 4. Discussion

Sarcopenia, a gradual muscle condition marked by the swift decline in muscle mass and functionality, is typically identified through the evaluation of muscle strength, commonly measured through grip-strength assessments [12]. Following the initial assessment of muscle strength, subsequent diagnosis involves the evaluation of muscle mass, predominantly estimated using DXA for lean mass. Additional tools like BIA, CT scans, and MRI are considered in specific cases [13]. Ongoing discussions among experts seek to enhance the definition of sarcopenia, particularly in terms of criteria related to physical performance [9,31]. The initial definitions of sarcopenia were predominantly centered on the evaluation of muscle mass, but contemporary focus has shifted to encompass the assessment of muscle function, encompassing both strength and power [32]. Acknowledging the intricacies of this stepwise diagnosis, our study adopted a retrospective approach utilizing TMT. Renowned for its robust correlation with both handgrip strength and skeletal muscle mass, TMT emerges as a dependable marker in assessing sarcopenia [17,18,19].

In this study, we enrolled a total of 28 participants to comprehensively investigate the relationship between muscle mass/strength and TMT. The demographic and clinical characteristics of the participants are outlined in Table 1, with a mean age of 38.1 years. Anthropometric measures, such as mean calf circumference (37.3 cm) and mean thigh circumference (43.5 cm), were systematically assessed, providing crucial data points for evaluating muscle mass. Handgrip strength, a representative measure of muscle strength, yielded a mean value of 28.7 kg, while the mean five-time chair stand test was determined to be 5.5 s. Additionally, TMT was measured, with a mean value of 9.7 mm. Our correlation analysis aimed to elucidate the interplay between muscle mass/strength and TMT. Moderately positive correlations were observed among the variables, demonstrating associations between calf circumference (r = 0.413, *p* = 0.029), thigh circumference (r = 0.486, *p* = 0.008), handgrip strength (r = 0.444, *p* = 0.018), and TMT (Figure 3). These findings underscore the interconnected nature of muscle mass and strength parameters with temporal muscle thickness in our study. Noteworthy is the absence of a significant correlation between the five-time chair stand test and TMT (r = −0.000, *p* = 0.998), suggesting that TMT may not be directly related with the five-time chair stand test.

Several methodologies are available for the measurement and assessment of muscle mass, strength, and performance within the field of clinical evaluation and research. The gold standards for assessing skeletal muscle mass, including CT, MRI, and DXA, are acknowledged for their precision; however, their utilization in community-based settings is constrained due to the substantial costs associated with equipment and the requirement for personnel with specialized skills [33]. In the 2019 guidelines set forth by the AWGS, the recommendation emphasizes the utilization of either DXA or multi-frequency BIA, both of which are to be adjusted for height, as the preferred methods for assessing muscle mass in the diagnostic process of sarcopenia [20]. The measurement of TMT serves as a noninvasive and straightforward assessment method, rendering it highly suitable for implementation in community settings. Moreover, it showcased a substantial positive correlation, with correlation coefficients of 0.81 in men and 0.73 in women, when compared to DXA-based appendicular skeletal muscle mass, underscoring its validity and relevance in the evaluation of muscle parameters [34]. Additionally, the measurement of TMT distinguishes itself from conventional methods used for evaluating muscle quantity. Its protocol is characterized by its simplicity, expeditiousness, reliability, independence from the patient’s phenotype, and the absence of a need for supplementary examinations. TMT can be acquired from T1-weighted MR images, with or without contrast enhancement, routinely obtained during cranial MR imaging. The application of predefined anatomical landmarks guarantees outstanding inter-rater reliability, a consistency noted not only in the current study, but also in previous research investigations. This underscores the robustness and reproducibility of TMT measurements, highlighting its potential as a valuable tool in clinical assessments [18,35]. Due to the fact that the measurement of TMT in a single patient takes approximately 30 s, swift assessments are feasible [18]. The observed association within this study between TMT and calf circumference implies a potential reflective relationship, suggesting that TMT may serve as an indicative measure of appendicular skeletal muscle mass within the context of our investigation. In various neurological disorders, TMT is recognized as a surrogate marker for sarcopenia. TMT has been associated with sarcopenia not only in cancers, such as squamous cell cancer of the head and neck, glioblastoma, and cerebral metastasis, but also in neurological conditions, such as cognitive function and amyotrophic lateral sclerosis [36,37,38,39,40]. In a preceding study targeting patients with ischemic stroke, the measurement of sarcopenia risk using the SARC-F questionnaire demonstrated independent associations with TMT assessed by CT [19]. The SARC-F questionnaire systematically assesses five distinct elements, comprising strength, walking assistance, standing up from a chair, ascending stairs, and occurrences of falls, providing a comprehensive framework for evaluating multiple facets of physical function and potential indicators of sarcopenia [30]. These linkages underscore the broad applicability and significance of TMT as an indicative marker across diverse health conditions and disease states. In the current study, specifically targeting individuals with a SARC-F score of 0, the observed substantial correlation of TMT with handgrip strength, calf circumference, and thigh circumference persisted, offering noteworthy insights even within the healthy individuals, thereby emphasizing the relevance of TMT as an indicator of muscle health across various measures of physical function.

Frequently utilized physical performance assessments encompass a range of established measures, such as the Short Physical Performance Battery (SPPB), normal walking speed, 6 min walk test, stair-climb power test, timed up-and-go test, and the five-time chair stand test, all contributing to a comprehensive evaluation of individuals’ functional capacities across various domains of physical performance [31,41]. The most frequently used test, usual gait speed, demonstrated a robust association with the initiation of disability and mortality [42,43]. However, gait speed poses the disadvantage of necessitating a sufficiently spacious area for the patient to walk, presenting a challenge in achieving precise measurements. The five-time chair stand time has been suggested as an alternative indicator of gait speed for the diagnosis of sarcopenia [44]. Although the older control subjects were categorized into age groups (60–69, 70–79, and 80–89 years), their five-time chair stand test scores did not exhibit a statistically significant difference compared with people with balance dysfunction [45]. The present study, conducted on healthy adults with a mean age of 38.1 ± 11.2 years, did not demonstrate a correlation between TMT and the five-time chair stand test used to assess physical performance. This aligns with findings from a previous study targeting patients with Parkinson’s disease, in which gait speed and TMT were also unrelated [46]. Given that diminished physical performance serves as a predictor for adverse outcomes and plays a crucial role in identifying the severity of sarcopenia, the observation that no association was found in this study between TMT and the five-time chair stand test in normal individuals suggests a lack of correlation between these variables. Therefore, it may be inferred that the relationship between TMT and the five-time chair stand test is contingent upon the presence of underlying conditions affecting physical performance, emphasizing the need for a nuanced interpretation of these metrics, especially in the context of healthy individuals. The observed correlations between TMT, handgrip strength, and calf and thigh circumference in the healthy individuals presented in this study may serve as a reference for future sarcopenia research utilizing TMT. The assessment of TMT proves to be feasible and can be seamlessly integrated into standard brain CT or MRI examinations, thereby presenting a distinct and valuable advantage within the diagnostic process. The outcomes derived from the findings of this study substantiate and lend robust support to the utilization of TMT as a reliable and predictive indicator for the presence of sarcopenia. This could facilitate retrospective research by calculating TMT using previously conducted MRI or CT scans in individuals with chronic illnesses, assisting in predicting outcomes related to sarcopenia. Furthermore, out devised diagnostic device, Allgeun, is foreseen to be as an economical and portable instrument, facilitating the diagnosis of sarcopenia within the confines of a conventional clinical setting.

This study has some limitations. Firstly, this study is limited by its single-center design and relatively modest sample size, which restricts its ability to generalize findings to a broader and more diverse population. Secondly, the cross-sectional nature of the study prevents the establishment of a definitive temporal relationship between TMT and the various components associated with sarcopenia. Thirdly, while the Allgeun device shows promise in its early stages of development, its widespread commercialization or adoption has yet to be achieved. The availability of Allgeun’s components for acquiring medical data may be limited despite their common usage. Furthermore, a significant limitation is that sarcopenia primarily affects the elderly population, while this study focused on a cohort of young and healthy individuals. The decline in muscle mass starting from approximately 40 years of age onwards, while assisting in establishing references for the healthy population, limits the generalizability of findings to the elderly [4]. Additionally, the study did not target populations with sarcopenia, in addition to mentioning only the elderly population. Lastly, the measurement of TMT by a single radiologist, despite their presumed substantial clinical experience, lacks cross-validation, posing a limitation to the study’s robustness. Despite these limitations, this study has several strengths. In contrast to preceding studies that primarily focused on TMT in patients with neurological diseases, our investigation uniquely explores its relevance in healthy, normal individuals. The initiation of muscle mass decline, commencing around the age of 40, underscores the significance of conducting research targeting younger individuals [4]. A distinctive strength lies in our direct comparison of TMT with the fundamental components crucial for diagnosing sarcopenia, encompassing muscle strength, mass, and physical performance. Moreover, our study establishes a compelling correlation between TMT and muscle strength and mass, leveraging the capabilities of Allgeun. This indicates the device’s suitability for the screening of sarcopenia, emphasizing its versatility through the utilization of the five-time chair stand-up test, a robust measure of physical capability. Consequently, Allgeun not only serves as an effective screening tool for sarcopenia, but also proves valuable in evaluating its severity.

## 5. Conclusions

Our research findings underscore the potential of TMT as a valuable indicator in the context of sarcopenia, specifically pertaining to the assessment of muscle mass and strength. Additionally, our study has effectively showcased the practical utility of our new device, Allgeun, in the screening and diagnostic evaluation of sarcopenia severity. Looking forward, as Allgeun undergoes further refinement and miniaturization in the process of product development, we envision that the screening for sarcopenia will seamlessly integrate into routine clinical practices. The anticipation of broader applicability hinges on the successful stabilization and downsizing of Allgeun. Subsequent larger-scale studies are imperative to validate the robustness of our present results and to establish the comprehensive effectiveness of Allgeun in the realm of sarcopenia assessment.

## Figures and Tables

**Table 1:** Clinical characteristics of the participants.

	Participants (N = 28)
Age, years	38.1 ± 11.2
Male, n (%)	10 (35.7)
Calf, cm	37.3 ± 4.0
Thigh, cm	43.5 ± 5.2
Handgrip strength, kg	28.7 ± 10.6
Physical performance, seconds	5.5 ± 1.3
Temporal muscle thickness, mm	9.7 ± 1.9

The data are presented as mean and standard deviation.

## Data Availability

The datasets generated during and/or analyzed during the current study are available from the corresponding author on reasonable request.
